# Blunt Tracheal Trauma Managed Conservatively and Surgically: Two Case Reports

**DOI:** 10.1155/cris/1502104

**Published:** 2026-04-18

**Authors:** Mohammad Alabdallat, Ahmad Kloub, Basil Younis, Ibrahim Afifi, Ayman El-Menyar, Ruben Peralta, Sandro Rizoli, Hassan Al-Thani

**Affiliations:** ^1^ Department of Trauma Surgery, Hamad Medical Corporation, Doha, Qatar, hamad.qa; ^2^ Department of Trauma Surgery, Clinical Research, Hamad Medical Corporation, Doha, Qatar, hamad.qa; ^3^ Department of Clinical Medicine, Weill Cornell Medicine, Doha, Qatar, qatar-weill.cornell.edu; ^4^ Department of Surgery, Universidad Nacional Pedro Henriquez Urena, Santo Domingo, 10100, Dominican Republic, unphu.edu.do

**Keywords:** conservative, road traffic injury, surgical, tracheobronchial injuries, trauma

## Abstract

Traumatic tracheobronchial injuries (TTBIs), while rare, are associated with significant morbidity and mortality. The management of these injuries requires a high degree of clinical suspicion, rapid diagnosis, and a multidisciplinary approach. We present two cases of TTBI, managed with varied approaches to achieve prompt, comprehensive, and optimal outcomes. The first case reported a 20‐year‐old male who presented with a blunt traumatic posterior tracheal wall at the T1/T2 level that was managed conservatively. The second case reported a 25‐year‐old motorcyclist presented with a tracheal injury at the T1 vertebra level that required surgical repair. The two treatment options were successful. The management of TTBI requires a highly individualized approach, balancing the urgency of surgical intervention with the potential benefits of conservative management.

## 1. Introduction

Traumatic tracheobronchial injuries (TTBIs) lead to significant morbidity and mortality. Due to the life‐threatening nature of blunt tracheobronchial injuries, doctors need to be more aware, keep a close eye on patients, and act quickly. This is especially important when there are critical injuries to multiple body regions, which can make it challenging to see the whole picture.

The incidence of TTBI is very low, at 0.4%. However, this rate could not be accurate, as most patients die before reaching the hospital [1]. Despite this low incidence, the potentially life‐threatening injury risk and high mortality rate (24.6%) highlight its importance [2]. It presents with variable clinical manifestations such as dyspnea, subcutaneous or mediastinal emphysema, hemoptysis, rib fractures, and Hamman’s sign (a mediastinal “crunch sound” during auscultation of the heartbeat) [2].

## 2. Case 1

A 20‐year‐old male motorcyclist was brought to the trauma room (TRU) as he was hit by a car. The patient had a Glasgow Coma Scale (GCS) score of 14 at the scene. However, upon arrival, he had decreased oxygen saturation levels; therefore, endotracheal intubation was performed to secure his airway.

**Figure 1:**
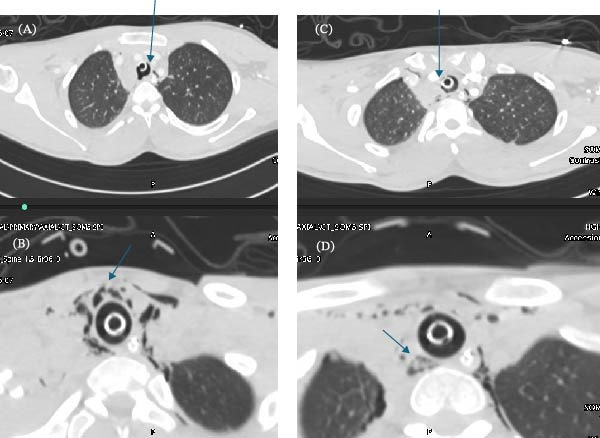
ETT in place (A, B) with posterior tracheal wall irregularity and surgical emphysema (C, D).

A pan CT scan revealed a bleed in the tentorial and subarachnoid areas, a small filling defect in the internal carotid artery inside the skull at the C1 level, a 2–4 mm irregularity in the posterior wall of the trachea at the T1/T2 level, a peritracheal hematoma, and surgical emphysema that extended into the upper mediastinum (Figure 1). He also had a small apical pneumothorax, contused lung, mild hemoperitoneum, Grade 1 liver, and Grade 1 splenic injury with no blush and an open tibia fracture.

The endotracheal tube (ETT) adequately bridged the tracheal injury, necessitating no further action at that time. The thoracic surgeon advised conservative management due to the patient’s stability and followed up with a chest X‐ray (CXR) after extubation. Flexible upper gastrointestinal endoscopy was performed to assess possible esophageal injury. Examination revealed a well‐circumscribed intramural hematoma located approximately 10 cm from the upper incisors, without evidence of mucosal laceration, perforation, or active bleeding. No contrast esophagography was performed, as endoscopy demonstrated intact esophageal continuity and no signs of transmural injury.

The patient remained intubated for airway protection during planned orthopedic interventions. On hospital day 9, following a multidisciplinary evaluation by the thoracic surgery team, he was extubated successfully. Given sustained clinical stability, additional investigations, such as bronchoscopy or repeat CT imaging, were not pursued. Postextubation, with close clinical observation, there was no evidence of airway compromise. He remained asymptomatic with stable respiratory function and was transferred to the rehabilitation center.

## 3. Case 2

**Figure 2:**
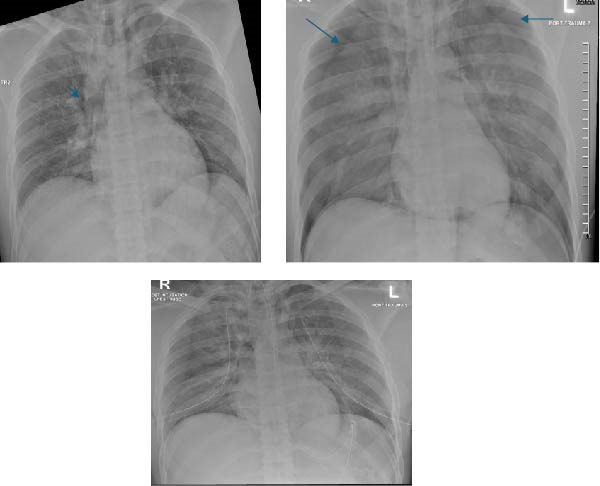
Pneumomediastinum in the initial chest X‐ray. The second chest X‐ray after 20 min revealed new bilateral pneumothorax.

A 25‐year‐old motorcyclist was brought to the emergency department after colliding with a fixed object. On initial assessment, the airway was patent and maintained; breathing was equal with bilateral air entry; oxygen saturation was 95% on room air; the patient was hemodynamically stable, with a GCS score of 15 and equal, reactive pupils. Focused assessment with sonography for trauma was negative. He complained of mid cervical spine pain and bilateral wrist pain with obvious deformities. An initial chest radiograph demonstrated a small pneumomediastinum without evidence of pneumothorax or hemothorax (Figure 2).

**Figure 3:**
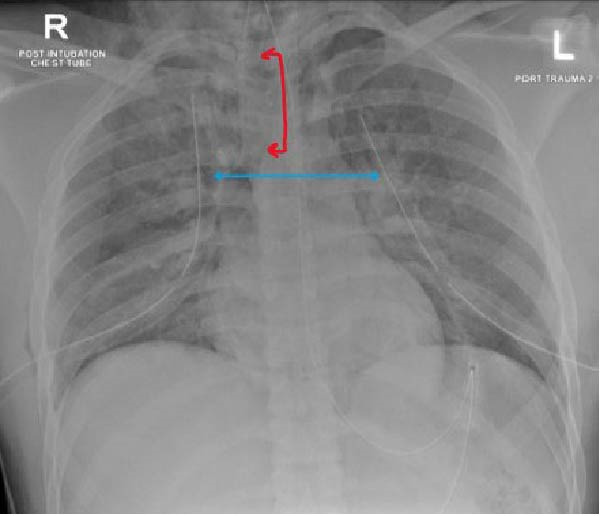
Bilateral chest tubes with high endotracheal tube level (in red).

During pan CT imaging and before contrast administration, the patient became anxious and developed acute respiratory distress with audible wheeze, and oxygen saturation decreased to 80% despite oxygen supplementation at 5 L/min via face mask. An initial working diagnosis of anaphylaxis was considered, and the patient received hydrocortisone, salbutamol nebulization, and subcutaneous adrenaline. A repeat chest radiograph subsequently demonstrated extensive bilateral pneumothoraces. Bilateral chest tubes were inserted (Figure 3); however, the patient’s oxygen saturation continued to deteriorate to below 50%, and he became less responsive.

Emergency endotracheal intubation was performed by the anesthesia team using a 7.5 mm ETT, inserted under direct vision with a stylet and secured at 22 cm at the teeth. Mechanical ventilation was initiated with an inspired oxygen concentration of 100%, a tidal volume of approximately 450 mL, a respiratory rate of 20 breaths/min, and a positive end‐expiratory pressure of 5 cm H_2_O, resulting in an improvement in oxygen saturation to 91%. A postintubation chest radiograph demonstrated the ETT tip positioned significantly above the carina. The tube was sequentially advanced to 23 cm and then to 26 cm at the teeth; despite this, repeat chest radiographs continued to show the tube positioned above the carina, and oxygen saturation stabilized at approximately 93%.

**Figure 4:**
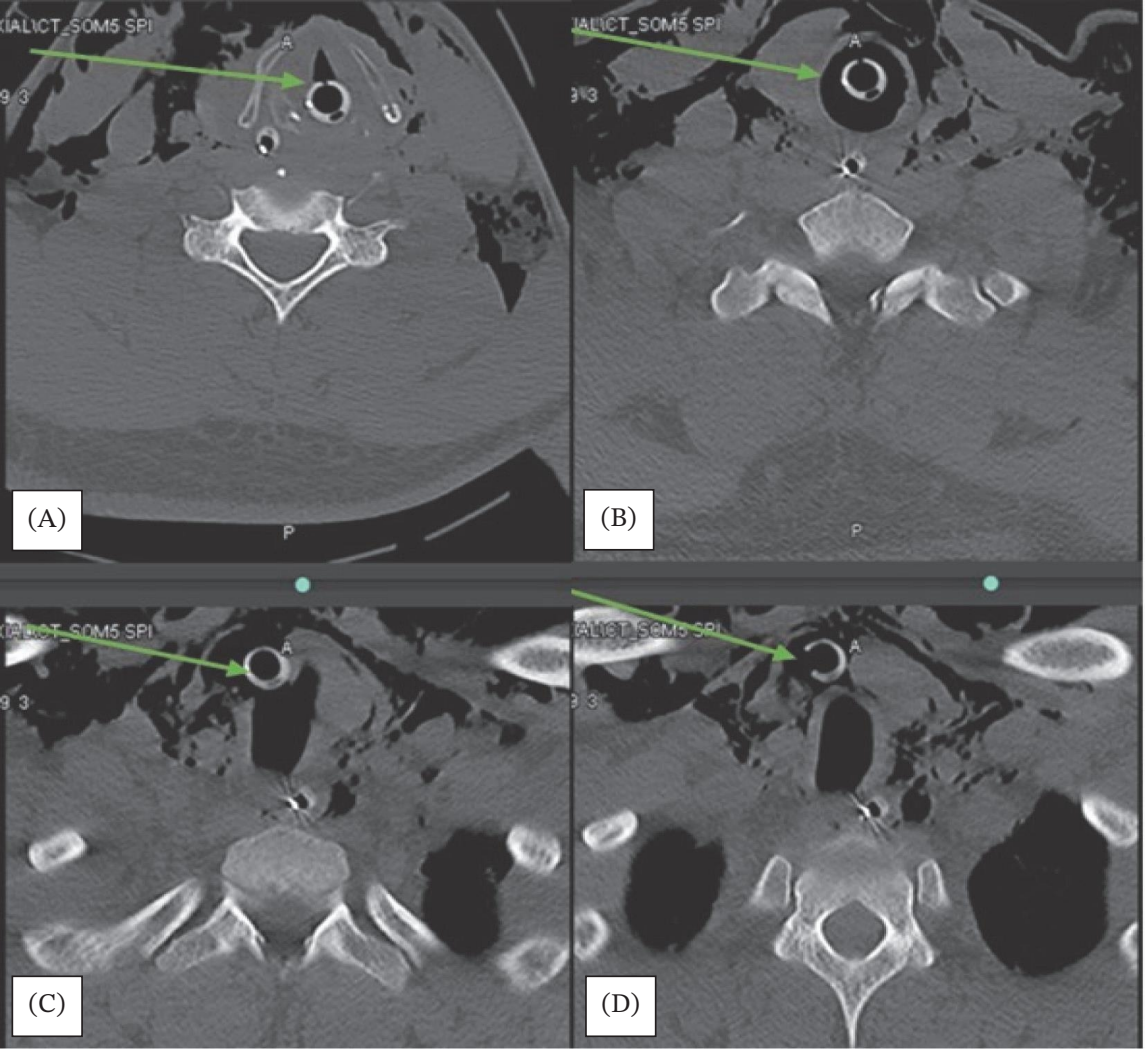
(A) Endotracheal tube (ETT) at the vocal cord level. (B) ETT below vocal cord. (C) ETT immigrates outside the trachea. (D) ETT orifice opened subcutaneously outside the trachea.

Flexible bronchoscopy was performed through the tracheal lumen. It demonstrated a soft tissue mucosal mass or hematoma at the distal end of the tube, with inability to visualize the carina, raising concern for tracheal injury. Following stabilization, whole body CT scan demonstrated anterior tracheal perforation at the level of the T1 vertebra, with the ETT having migrated extratracheally into the lower anterior cervical subcutaneous tissues (Figure 4). Extensive surgical emphysema was present, extending from T11 to the neck and face. Associated injuries included a fracture of the right posterior arch of C1, multiple fractures involving the transverse processes and foramen transversarium from C5 to T1, and a fracture of the left articular process of L5.

**Figure 5:**
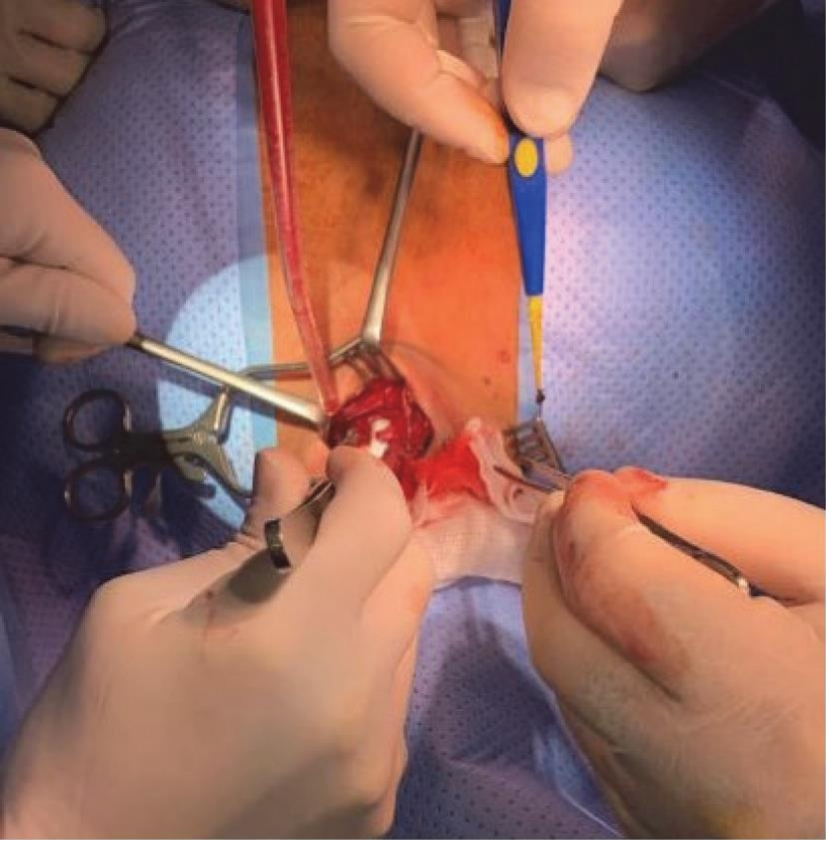
Surgical closure of the tracheal defect.

The patient was transferred to the operating theater, where fiber optic bronchoscopy confirmed extrusion of the ETT through an anterior tracheal perforation at the T1 level, with disruption of a tracheal cartilage ring. The posterior membranous trachea remained intact, and no significant vascular injury was identified. Open surgical repair was performed by the thoracic surgery team via an anterior cervical approach, with primary closure of the anterior tracheal defect using 4‐0 polydioxanone sutures. Ventilation was maintained with conventional endotracheal ventilation throughout the procedure, with extracorporeal membrane oxygenation available on standby but not required. Following repair, the ETT was repositioned under direct visualization (Figure 5).

Postoperatively, the patient was admitted to the trauma intensive care unit and remained mechanically ventilated overnight. He was extubated on postoperative day one with a GCS score of 15, and the chest tubes were subsequently removed. He was transferred to the trauma ward and later underwent definitive orthopedic surgery for bilateral wrist fractures. The patient remained clinically stable and was discharged without hoarseness, respiratory compromise, or neurological deficit. Routine postoperative bronchoscopy was not performed because the patient remained stable and showed no clinical or radiographic evidence of airway compromise during hospitalization or follow‐up.

## 4. Discussion

Traumatic tracheal injuries have substantial mortality, therefore, early diagnosis and treatment is mandatory including conservative management for minor laceration or immediate surgical intervention and stenting for significant injury (i.e., >2 cm) [2, 3]. Missed TTBI injuries are expected, especially in the presence of coexisting life‐threatening vital organ injuries. After a month, granulation tissue forms and potentially leads to stenosis, which manifests as stridor and dyspnea, while bronchial stenosis manifests as wheezing and postobstructive pneumonia. CXR, CT, and bronchoscopy have become functional modalities for diagnosis [3]. In the first case, we highlight the critical role of conservative management in treating blunt tracheal injuries, particularly when the injury measures 2–4 mm. We avoided the need for immediate surgical intervention by using an ETT to bridge the injury, demonstrating the effectiveness of a conservative approach in select cases. A conservative approach is taken for tears less than 2 cm [4].

Nonoperative management of TTBI may be appropriate in carefully selected patients who are hemodynamically stable, maintain adequate respiratory function with or without ventilatory support, show no evidence of sepsis, and have short airway ruptures, particularly when diagnosis is delayed. Additional factors favoring conservative treatment include the absence of associated esophageal injury, minimal and nonprogressive pneumomediastinum, and limited subcutaneous emphysema [5].

This approach is often more applicable to iatrogenic tracheobronchial injuries. For a posterior tracheobronchial tear, conservative treatment may be appropriate if there is minimal, nonprogressive pneumomediastinum, no mediastinitis, the ability to evacuate pneumothorax without a persistent large air leak, no esophageal injury, the ability to maintain ventilation, and the ability to position the endotracheal cuff away from the injury site with ventilation feasible under minimal positive end‐expiratory pressure [4].

In the second case, we highlight the importance of early surgical intervention in the management of tracheal injuries. The decision to proceed with early surgical repair was crucial in achieving a favorable outcome. Surgical repair of the tracheal injury stabilized the patient’s respiratory status and facilitated a smooth postoperative recovery.

The risk of airway obstruction, significant air leaks, and the potential for mediastinal infection generally guide the decision to perform surgical repair of an acute tracheobronchial tear. Surgical intervention is commonly preferred when the tracheal tear exceeds 4 cm. Emergency surgery is necessary if there is esophageal prolapse into the tracheobronchial lumen, ventilation difficulties, or tracheobronchial injury identified during surgery [4]. The optimal management of intermediate‐length injuries (~2–4 cm) remains uncertain and should be individualized, taking into account airway anatomy, clinical status, patient comorbidities, and available local expertise [5].

Early surgical intervention is often necessary for successful outcomes in managing complex polytrauma cases involving significant tracheal injuries.

A previously published case from our center is summarized for comparison, highlighting the risks of delayed diagnosis and the use of tracheal stenting as a temporary management strategy [6]. Table 1 summarizes the presentation and management of our cases, along with a prior case from our center. It is not a part of the current two‐case report. Stenting played a crucial role in managing the patient’s tracheal stenosis, which developed following an overlooked tracheal transection. In this scenario, a tracheal stent is used to maintain airway patency, prevent restenosis, and facilitate tissue healing. Stenting is a good option in cases where surgical resection is either not feasible or associated with high risks, particularly in patients with complex or recurrent stenosis. The stent provided a temporary solution, enabling the patient to be extubated and discharged home in stable condition.

**Table 1:** Patient demographics and injury details.

Sex	Age	Mechanism of injury	Location of injury	Size of injury	Time of diagnosis	Treatment	Outcome
Male	20	Motorcyclist hit by a car	Posterior tracheal wall at T1/T2 level	2–4 mm	Less than 6 h	Conservative management with endotracheal tube bridging	Stable recovery, extubated, no residual complications
Male	25	Motorcyclist collided with object	Tracheal injury at T1 vertebra	2–4 cm	Less than 6 h	Surgical repair with PDS 4–0	Successful recovery, no respiratory complications
Male^a^	29	Pedestrian hit by a car	Complete transection 2.5 cm proximal to carina, anterior wall	Complete transection	Delayed (postdischarge)	Stenting and multiple bronchoscopic interventions with ECMO	Complications during stent removal, fatal outcome [6]

^a^A previously published case from our center and not part of the present series, cited for purpose of comparison.

However, the use of tracheal stents is not without complications. Complications of stents include migration, fracture, infection, biofilm formation, and obstruction due to defective mucus clearance or granulation tissue [7]. In this case, the patient developed granulation tissue around the stent, which led to recurrent obstruction and respiratory distress. Granulation tissue formation is a known complication of tracheal stenting, often necessitating further intervention to maintain airway patency. This case demonstrates the need for close follow‐up and timely management of stent‐related complications, including the potential need for stent removal and replacement.

The patient’s representation with stridor and respiratory distress 1 month postdischarge highlights the long‐term challenge of managing tracheal injuries.

The development of granulation tissue and the subsequent difficulties encountered during stent removal, which can lead to fatal complications, illustrate the high risks associated with this approach. The decision to place a tracheal stent must be carefully considered, weighing the benefits of airway stabilization against the potential for severe complications, such as granulation tissue formation and restenosis. In both cases, the absence of clinical findings during follow‐up period extending up to 3 months makes the development of clinically significant posttraumatic tracheal stenosis unlikely. Therefore, no additional imaging or bronchoscopy was indicated. The multidisciplinary approach and preparation before the stent replacement should be considered [6]. Table 2 summarizes the timeline for the previously published case.

**Table 2:** Case 3 timeline^a^.

Phase	Key events	Airway/ECMO	Outcome
Initial trauma	MVC; desaturation; bilateral pneumothoraces; multiple chest tubes placed	Intubated	Stabilized; discharged
Readmission	SOB; CT reconstruction revealed missed tracheal transection; bronchoscopy showed tight stenosis just above carina	—	Admitted with type 2 respiratory failure
First intervention	Rigid bronchoscopy → hypoxia	ETT #6 dilation; VV‐ECMO initiated	Stabilized
Second intervention	Further dilation	ETT #7.5; ECMO continued	—
Extubation phase	No residual stenosis by day 9	Extubated; ECMO discontinued	Stable
Restenosis episode	Worsening respiratory status; restenosis	ETT #8 dilation; covered stent placed; ECMO standby	Extubated; discharged
One‐month return	Stridor; granulation obstructing distal trachea	ETT #7.5 passed; stent embedded in granulation	Planned stent exchange
Terminal event	Stent removal → immediate hypoxia → cardiac arrest; rescue attempts failed	Manual airway attempts failed; two small ETTs placed into bronchi; ECMO unavailable	Died intraoperatively

^a^A previously published case from our center and not part of the present series, cited for purpose of comparison [6].

## 5. Conclusion

The management of TTBI requires a highly individualized approach, balancing the urgency of surgical intervention with the potential benefits of conservative management. These cases underscore the importance of early recognition, rapid diagnostic workup, and the integration of a multidisciplinary team to optimize outcomes. While conservative management may be appropriate for small tracheal tears under specific conditions, surgical repair becomes essential in more extensive injuries to prevent life‐threatening complications. Additionally, the use of tracheal stents, while potentially life‐saving, carries significant risks and requires careful consideration, monitoring, and timely intervention to address complications.

## Author Contributions

All authors contributed substantially to this manuscript.

## Funding

The authors have nothing to report.

## Disclosure

All authors have read and approved this manuscript for submission.

## Ethics Statement

Ethical approval was obtained from the Institutional Review Board (MRC‐04‐24‐722) at the Medical Research Center, Hamad Medical Corporation (HMC), Doha, Qatar. The Medical Research Center (MRC) granted a waiver of consent for publication, provided that no photo or personal identifiers were included.

## Consent

No written consent has been obtained from the patients as there is no patient identifiable data included in this case report.

## Conflicts of Interest

The authors declare no conflicts of interest.

## Data Availability

Data sharing does not apply to this article as no new data were created or analyzed in this study.
